# 

**Published:** 1882-11

**Authors:** 


					﻿NOTICE.
Ml articles for publication, books for review, business communications, etc.,
must be sent to Editor, 2109 Chestnut Street, Philadelphia.
attention I
“Resolved, That it is the opinion of the publishers of The Hlomceopathic
Physician that no journal can properly sustain the responsibilities or fulfill,
all. the.duties: of. its position unless it ehjoys absolute freedom from debt and
unrestricted 'receipt pf its subscription?, as' provided for in the code of the
U.. 8. Postal Regulations.” , (Passed withouta dissenting Voice !)
The above resolution refers entirely to the consultation question! It is to
be hoped that delinquent subscribers will consult their earliest convenience and
pay their dues. .
Authors of accepted papers are furnished with copies of the
number in which their articles appear V application for such copies
to be ma<|e when sending papers. - Any irregularity in the receipt
of this Journal should be promptly reported to Editor.
				

